# Oxidative stress and pediatric diabetic cardiovascular complications: emerging research and clinical applications

**DOI:** 10.1152/ajpheart.00673.2024

**Published:** 2025-02-28

**Authors:** Saman Saedi, Yi Tan, Sara E. Watson, Joshua D. Sparks, Kupper A. Wintergerst, Lu Cai

**Affiliations:** 1 Department of Animal Science, College of Agriculture, Shiraz University, Shiraz, Iran; 2 Pediatric Research Institute, Department of Pediatrics, University of Louisville School of Medicine, Louisville, Kentucky, United States; 3 Wendy Novak Diabetes Institute, Norton Children’s Hospital, Louisville, Kentucky, United States; 4 Department of Pharmacology and Toxicology, University of Louisville School of Medicine, Louisville, Kentucky, United States; 5 Norton Children’s Endocrinology, Department of Pediatrics, University of Louisville School of Medicine, Louisville, Kentucky, United States; 6 Division of Pediatric Cardiology, Department of Pediatrics, University of Louisville School of Medicine, Louisville, Kentucky, United States; 7 Center for Integrative Environmental Health Sciences, University of Louisville School of Medicine, Louisville, Kentucky, United States; 8 Department of Radiation Oncology, University of Louisville School of Medicine, Louisville, Kentucky, United States

**Keywords:** cardiovascular complications, diabetic cardiomyopathy, oxidative stress, pediatric diabetes, therapeutic strategies

## Abstract

The prevalence and incidence of diabetes in pediatrics have dramatically increased over the last three decades. Comparatively, pediatric diabetes has faster pancreatic β-cells decline and early progression to complications compared with adult diabetes. Therefore, diabetic complications are a major concern in children and adolescents with diabetes. Diabetes has detrimental effects on the macro- and microvascular systems, resulting in cardiovascular diseases, leading causes of morbidity and mortality in youth with diabetes. Oxidative stress plays a critical role in developing cardiovascular complications in the context of pediatric diabetes. In pediatric patients with diabetes, several factors can contribute to the development of excess reactive oxygen species and oxidative stress, including nutritional deficiencies, puberty, environmental exposures, and metabolic disorders such as obesity and high blood pressure. The present study aims to raise awareness of diabetic cardiovascular complications in children and adolescents with diabetes and the role of oxidative stress and their molecular mechanisms in the pathogenesis of cardiovascular complications. In addition, some novel therapeutic strategies for the treatment and prevention of diabetic cardiovascular complications in the pediatric populations are highlighted. In summary, children and adolescents with diabetes no matter type 1 diabetes (T1D) or type 1 diabetes (T2D), have many features similar to those in adults with same kinds of diabetes, but also have many their own features distinct from adults. By developing targeted therapies and preventive measures, healthcare providers can better address the rising incidence of diabetes-related complications in children and adolescents.

## INTRODUCTION

Diabetes is a group of metabolic disorders caused by abnormal carbohydrate metabolism, insufficient insulin production, and/or insulin resistance resulting from environmental and genetic components (1). The two most common classifications of diabetes are type 1 diabetes (T1D) and type 2 diabetes (T2D), which have unique pathophysiological foundations, risk factors, and clinical manifestations (2). Individual metabolic lesions leading to pancreatic β-cells destruction in patients with diabetes result in an underlying relative or absolute insulin deficiency in T1D, whereas variable peripheral resistance to the effects of insulin or insufficient insulin production is seen in T2D (3). Although the prevalence of diabetes has dramatically increased over the last three decades, the early diagnosis and classification of diabetes are often difficult in practice (4). According to the International Diabetes Federation Diabetes Atlas, the global prevalence of diabetes was 536.6 million people aged between 20 and 79 yr in 2021, and this number is expected to reach 12.2% (783.2 million) by the year 2045 (1). The total estimated cost of diagnosed diabetes in the United States in 2022 is $412.9 billion, and the cost category analysis showed that care for people diagnosed with diabetes accounts for 1 in 4 healthcare dollars spent in the United States, 61% of which are attributable to diabetes. Based on the 2022 data, this inflation-adjusted direct medical costs of diabetes increased by ~7% from 2017 and 35% from 2012 calculations and will be continually increased in the following decades (5). The statistics above emphasize the importance of comprehending the mechanisms that contribute to the diabetes epidemic and the extensive impact it has on healthcare systems and economies (6).

In addition to the detrimental effects of diabetes on the microvascular system such as diabetic retinopathy and neuropathy, diabetes also has detrimental effects on the macrovascular system such as cardiovascular diseases (CVDs), which are the leading causes of morbidity and mortality in individuals with diabetes (7, 8). CVDs, including coronary artery disease (CAD), stroke, and peripheral arterial disease (PAD) mostly develop in patients with diabetes (9). Cardiovascular complications are also a major concern in children and adolescents with diabetes, particularly for T1D. This begins early in life, with a high prevalence of cardiovascular complications in infants of diabetic mothers: 18.7 and 46.9% of which had congenital heart disease and hypertrophic cardiomyopathy (10). A review has highlighted the T1D as a major risk factor for CVDs, causing both systolic and diastolic cardiac dysfunction in children (11). In addition, emerging research indicates that the presence of additional autoimmune diseases, such as celiac disease or Hashimoto’s thyroiditis, may further increase the risk of cardiovascular complications in young patients with T1D (12). This risk may be mitigated in part by the use of continuous glucose monitoring, insulin pumps, and hybrid closed-loop systems, which have been shown to improve glycemic control and reduce the risk of long-term complications, including CVDs (13).

Excess production of reactive oxygen species (ROS) or reactive nitrogen species (RNS) from metabolic abnormalities disorder and altered redox signaling in cells and tissues results in increased cellular oxidative stress. Oxidative stress plays a critical role in developing macro- and microvascular complications in pediatric diabetes (14, 15). Oxidative stress disrupts cellular function in multiple ways including mitochondrial dysfunction, altered molecular pathways, deregulation in cell cycle control, cell death, inflammation, and impairment of the antioxidant systems not only in β-cells, leading to its dysfunction or death but also in the insulin-dependent and insulin-independent peripheral tissues, resulting in systemic insulin resistance and chronic damage and dysfunction (14, 16, 17). All these cellular changes lead to the initiation of diabetes and the development of cardiovascular complications (14-16, 18). Therefore, a better understanding of the underlying mechanisms by which oxidative stress and associated pathogenic consequences promote cardiovascular complications in pediatric diabetes is crucial to developing more effective, targeted treatment strategies beyond just improved glycemic control.

Therefore, the current study will summarize the current status of children and youth with diabetes and their complications with a focus on the oxidative stress and its association with pediatric diabetic cardiovascular complications in the research and clinical applications. To this end, we collected the relevant articles written in English from PubMed and Google Scholar. Although the search was done without publication year restriction until January 22, 2025, the selected literature is predominantly from the 2000s to the present with a focus on new insights and advances, specifically, articles pertaining to oxidative stress and its potential role in the pediatric diabetic cardiovascular complications, as well as the implication in the pediatric population. For these ends, the current study used multiple key words alone or combined in diverse ways. The key words included three main terms: “pediatric diabetes,” “children with diabetes,” and “youth with diabetes,” and other secondary key words that included “complications,” “cardiovascular diseases,” “oxidative stress,” “mechanistic feature,” “insulin signaling,” “therapy or treatment.” In addition, the current study also followed the link of “similar articles” under certain typical single publication to trace more relevant articles. All cited articles were ready by more than three authors, and clinical concepts and conclusions were reviewed and confirmed by the coauthors in pediatric cardiology and endocrinology.

## DIABETES IN PEDIATRICS: A GROWING CONCERN

Although there is an overlap in the features of cardiovascular complications in adults and youth with diabetes, it is important to consider the pathophysiology and epidemiology that are unique to younger patients. This section will discuss certain features of diabetes and its cardiovascular complications found in children and teenagers with diabetes. The incidence and prevalence of diabetes vary by gender, age, race, ethnicity, and geographic location (3). T1D is the most common form of diabetes in youth, although there are more adults living with and diagnosed with type 1 diabetes (19). The number of children diagnosed with T1D has also been growing at an annual rate of ~3% (20) in Europe and the United States (21). Over the past 20 years, the prevalence of T2D in the children and youth population has also risen worldwide (22), particularly in the United States (23, 24). Most recently, the coronavirus disease 2019 (COVID-19) pandemic brought increased attention to children with pre-existing disorders such as diabetes (25) and an increase in the incidence of both T1D and T2D (26-28).

### Updated Prevalence and Incidence of Diabetes in Pediatrics

The overall prevalence of T1D has been gradually rising ~3–5% annually worldwide, with significant geographic variation (29-32). Historically, a World Health Organization 10-yr program, Multinational Project for Childhood Diabetes (Diabetes Mondiale or DIAMOND), investigated and characterized global incidence and mortality for T1D by collecting population-based data concerning T1D in >90 centers in 50 countries worldwide during 1990–1999 (33). Consequently, this program collected and published data on 75.1 million children (≤14 yr of age) with a total of 19,164 cases identified as having a diagnosed as T1D (34). In 2006, an updated report included a total of 43,013 cases of T1D out of 84 million children from 112 centers in 57 countries (32). The overall age-adjusted incidence rates of T1D varied from 0.1/100,000 in Zunyi, PR China, to 36.5/100,000 (34) or 40.9/100,000 (32) in Finland, with variations by age and sex, showing slightly higher in boys than in girls and lowest in children aged 0–4 yr and highest in youths aged 10–14 yr. Similarly, the T1D incidence of children of 0–14 of age from 1996 to 2022 demonstrated an increasing trend as 26.1/100,000 in a separate Germany study (35) with the similar variations of age and sex to those in Finland (32, 34).

**Figure 1.**
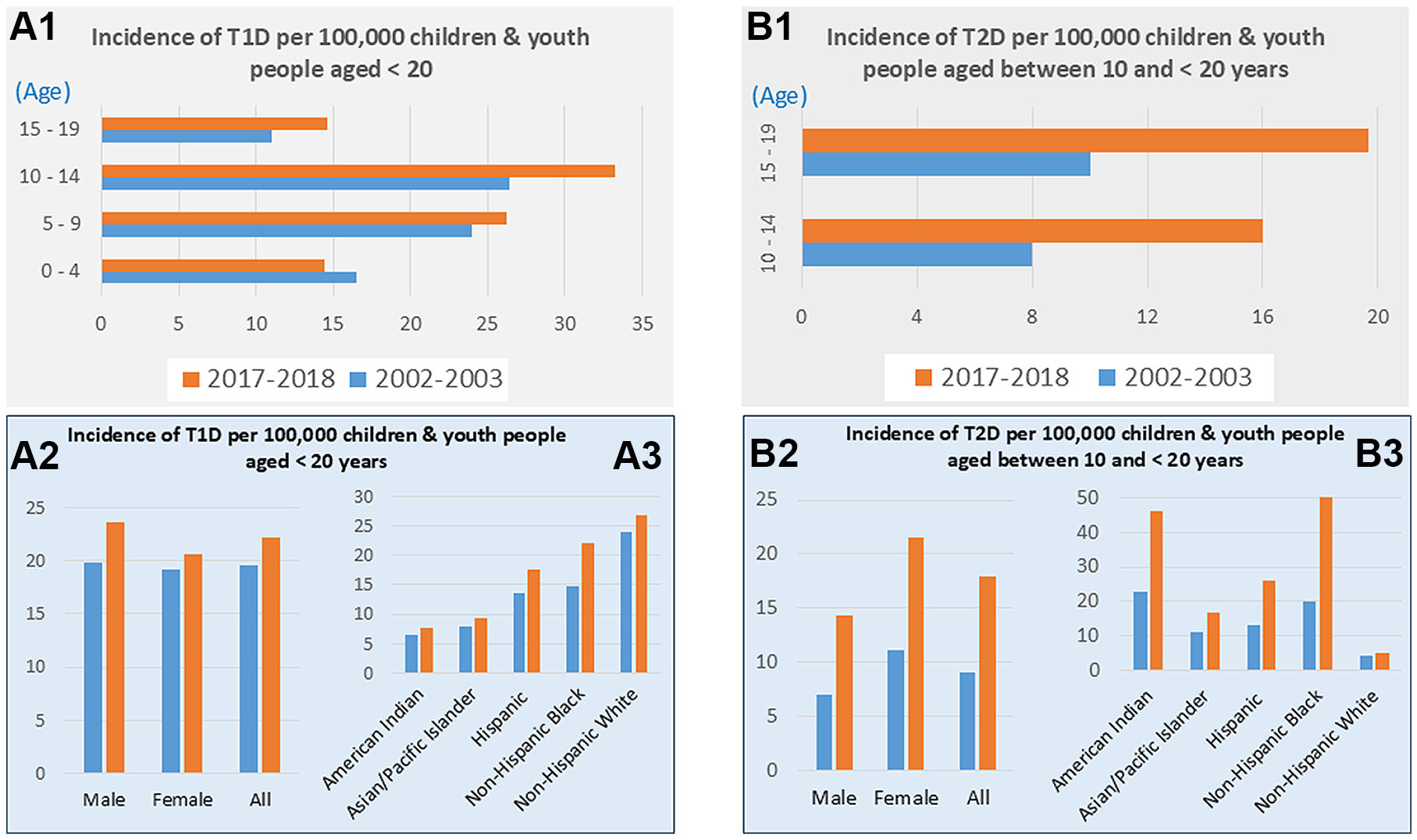
The prevalence of children and young people with T1D or T2D. The graph was made based on the data published by Wagenknecht et al. (36). *A1*, *A2*, and *A3* illustrate the incidence of T1D per 100,000 children and youth people at different age range (*A1*), different gender (*A2*) and different races (*A3*), respectively. *B1*, *B2*, and *B3* illustrate the incidence of T2D per 100,000 children and youth people at different age range (*B1*), different gender (*B2*) and different races (*B3*), respectively. Note: “All” in *A2* and *B2* means combination of males and females.

In North America, the prevalence was relatively high, ranked in the top 10 with 11/100,000 (United States) to the top 6 with 25/100,000 (Canada). Among these populations, the incidence increased with age and was the highest among children 10–14 yr of age (34), similar to those (35). Figure 1, based on the most recent SEARCH for Diabetes in Youth study by Wagenknecht et al. (36), shows that the prevalence of children and youth people with T1D at different ages (<20 yr old) during the time period 2017–2018 (brown bars) is higher than the prevalence during 2002–2003 (green bars) except for ages 0–4 yr old (Fig. 1A1). The increased trend is predominantly in the boys (Fig. 1A2). Recently the SEARCH for Diabetes Youth Study also estimated the increase from 185,000 in 2017 to 191,000 youths with T1D in 2060 with a relative increase in 3% (37), similar to those from 1996 to 2022 in Germany (35).

Although diagnosed T2D was rare in the adolescent population in the past, age now is no longer the most important differentiating feature between T1D and T2D. Figure 1B clearly indicates the increased prevalence of youth or adolescents with T2D in the eased (2010s and 2020s) (38-40): *1*) T2D mainly occurs in the youths aged 10–19 yr; *2*) compared with 2002–2003, the prevalence of youth with T2D are almost double in 2021–2028. The latter was consistent with the historical report by Lawrence et al. (38): the estimated T2D prevalence per 1,000 youths aged 10–19 yr increased significantly from 0.34 in 2001, to 0.46 in 2009, and to 0.67 in 2017, an absolute increase of 0.32 per 1,000 youths and a 95.3% relative increase over 16 yr. As predicted for youths with T1D, the number of youths with T2D was also predicated significant increase from 28,000 in 2017 to 48,000 in 2060 if the incidence remains constant as observed in 2017, with a relative increase in 69% for T2D (37).

Few cases of T2D were identified in children under the age of 10 yr, according to SEARCH reported in 2002–2012 (24). For those aged 15–19 yr, a higher incidence of T2D than T1D was observed (24). In alignment with this result, a Tokyo study showed that the incidence of T2D in children aged 13–15 yr was about six times higher than that in children aged 7–12 yr (41, 42). According to this evidence, the prevalence of T2D is more common among children in the age group between 10 and 19 yr, corresponding to the hormonal dynamics of puberty (43, 44). During puberty, there is a surge in growth hormone (GH) and insulin-like growth factor-I (IGF-1), which increases insulin resistance (45), leading to increased lipid breakdown and increased free fatty acids (FFAs) in the circulation. Besides, an increase in sex hormones during puberty, especially androstenedione, increases the acute insulin response, which is an independent predictor of T2D (46, 47).

Discussions outlined earlier indicate that the significant trend of children or young people with either T1D or T2D is globally increasing; however, it was significantly different among different racial and sex populations (Table 1; Fig. 1) and different countries (Table 1) for both T1D and T2D (72, 73). For instance, the prevalence of youths with T1D among non-Hispanic White is ~4–5 folds of American Indian, but the prevalence of youths with T2D is the lowest in non-Hispanic White compared with others (Fig. 1A3; Table 1). As shown in Fig. 1, A2 and B2, boys have a higher prevalence of T1D than girls but a lower prevalence of T2D than girls. In terms of geographic difference, a recent systematic review of literature on T2D incidence among children and adolescents from 25 countries revealed 41,600 new instances of youth-onset T2D worldwide in the year 2021, with the highest prevalence in PR China (734 cases/100,000 population), India (397/100,000), and the United States (285/100,000) and the lowest in Pakistan (88/100,000) (72).

### Diabetic Cardiovascular Complications in Pediatrics

A large body of evidence suggests that individuals with the early onset of T1D and T2D have prolonged exposure to the associated metabolic abnormalities, resulting in amplified long-term cardiovascular complications. Therefore, childhood and adolescence are critical periods to monitor for the onset of cardiovascular risk factors (74). The younger an individual is at diagnosis of T1D, the higher the risk of atherosclerosis and CVD (75). Rawshani et al. (76) reported that individuals with onset of T1D before 10 yr of age had ~11 times higher risk of atherosclerotic and CVDs compared with matched control participants, whereas girls with disease onset before 10 yr of age had ~13 times increased risk of atherosclerotic and CVD. The risk of acute myocardial infarction also was ~30 times higher in patients diagnosed with T1D under the age of 10 yr (76).

A study on clinical outcomes of patients with T2D (*n* = 354) and T1D (*n* = 470) with an onset age between 15 and 30 yr with a median follow-up of 21.4 and 23.4 yr, respectively, revealed a significantly higher mortality in T2D cohort with a twofold mortality compared with those with T1D. Total death and cardiovascular deaths for T2D occurred significantly earlier and more than T1D after diabetes onset at a relatively young age. They found that even with equivalent glycemic control, the prevalence of albuminuria remained high and the favorable cardiovascular risk factors were lower in the T2D cohort soon after diabetes onset. Macrovascular complications were also increased in patients with T2D. Therefore, this study suggests that young-onset T2D is the more aggressive phenotype of diabetes and is associated with greater mortality, more complications, and unfavorable cardiovascular disease risk factors when compared with T1D (77). This was also supported by the later study (78); however, the reason for the high incidence of T2D complications in young adults is not well understood but may be related to insulin resistance and a rapid worsening of β-cell function (79).

Cardiovascular complications in children and youths with diabetes contain diabetic cardiomyopathy (DCM) and accelerated atherosclerosis. DCM is when ventricular dysfunction occurs in patients with diabetes in the absence of CAD and hypertension (14, 80). DCM is caused by a combination of metabolic disorders, myocardial fibrosis, impaired energy utilization, small vessel disease, cardiac autonomic neuropathy, and insulin resistance. An early study with 40 teenagers aged 12–18 yr with T1D and 20 matched controls revealed all individuals with T1D had normal left ventricle (LV) dimensions, mass index, and systolic functions, but 25% of individuals with T1D had LV and right ventricle (RV) diastolic dysfunction, and 52.5% of individuals with T1D had delayed myocardial relaxation compared with controls, diagnosed by conventional Doppler (81). In the following study with asymptomatic T1D children with a mean age of ~9 yr (82) and ~11 yr (83), subclinical LV and RV systolic and diastolic dysfunctions even within a short duration of diabetes were also noticed, and poor glycemic control was associated with early subclinical LV systolic and diastolic impairment (83). However, it should be mentioned that for children with T1D, alterations in LV myocardial function could not be easily detected either clinically or by conventional echocardiography, but could be detected with tissue Doppler and speckle tracking (84). Moreover, concentric LV hypertrophy is noted to be common among adolescents with diabetes, especially in those with obesity, and is identified by the increase in left ventricular wall thickness over time (85).

Relatively less information on the features of DCM in children and adolescents with T2D is available. Echocardiographic examination for T2D Adolescents and Youth (TODAY) with an average of age 18 yr, revealed that adolescents with T2D have adverse measures of cardiac structure and function positively related to body mass index (BMI) and blood pressure (86). Moreover, concentric LV hypertrophy is common among adolescents with diabetes, especially in those with obesity, and is identified by the increase in left ventricular wall thickness over time (85). However, direct comparisons of youths with T2D (average age of ~24 yr) and those with T1D (average age of ~21 yr) with a median diabetes duration of ~12 yr revealed that participants with T2D had greater LV mass index and worse diastolic function compared with participants with T1D (87).

Atherosclerosis is a progressive disease that starts in childhood, and subclinical CVD may be present in youth within 10 yr of diagnosis with T1D (88). A cohort of patients with diabetes with a mean age of 15.6 yr showed higher levels of inflammatory biomarkers than their healthy peers, particularly the subgroup with T2D, compared with that with T1D (89). Obesity and T2D in pediatrics are characterized by a state of systemic chronic low-grade inflammation that triggers insulin resistance, oxidative stress, and endothelial dysfunction, which lays the basis for early accelerated atherosclerosis (90).

Recent studies have reported that young patients with T1D have delayed or reduced brachial artery flow-mediated dilatation reactivity and higher carotid-femoral pulse wave velocity (PWV), compared with healthy controls (91, 92). These findings are consistent with arterial stiffness. This peripheral arterial dysfunction might be a contributing cardiovascular risk factor. For instance, pulse pressure, which reflects arterial stiffness, has been reported to increase at a younger age in T1D as compared with healthy controls (93). A recent study has demonstrated arterial stiffness in the small resistance arteries to be independently associated with all-cause mortality and a composite of cardiovascular and/or diabetes-related mortality in T1D (94). On the contrary, in young patients with T1D, arterial stiffness is positively associated with glycated hemoglobin levels, disease duration, and insulin resistance (95). T2D has been identified as a major risk factor for accelerated vascular aging, measured as PWV or augmentation index (96, 97). Approximately one in four youth with T2D has a microvascular complication or hypertension at diagnosis, and the cumulative prevalence rises to >60% in 10–12 yr (98). These findings support accelerated vascular aging in youth-onset T2D and emphasize the need to identify the modifiable mechanistic drivers of disease progression (99).

### Pathophysiology of Diabetic Cardiovascular Complications in Pediatrics

Despite the exponential increase in the number of preclinical and clinical studies on diabetic cardiovascular complications over the past decades, pathophysiological mechanisms of cardiovascular complications associated with diabetes have not been as extensively evaluated in children as in adults. In the SEARCH for Diabetes in Youth study, youth with T2D exhibited a more atherogenic lipid profile compared with youth with T1D. This profile demonstrated low low-density lipoprotein cholesterol (LDL-C), higher total cholesterol (TC) and triglycerides (TG), and lower high-density lipoprotein cholesterol (HDL-C) (100, 101). Dyslipidemia, therefore, has been reported as an important target for the prevention of CVD in youths with diabetes. Consequently, both the American Diabetes Association (ADA) and the American Academy of Pediatrics (AAP) have recommended screening of dyslipidemia in youth with new-onset diabetes once glycemic control is established or 3 mo after commencing medication and annually thereafter (102, 103). In addition, diabetic ketoacidosis, as the most common acute complication in children, also contributes a great risk for a wide range of life-threatening complications in children and adolescents with T1D (104).

Although insulin resistance can take place in both T1D and T2D children and adolescents, its pathophysiology seems to be different in youth with T1D, as compared with those with T2D (105). The development of insulin resistance and T2D leads to increased FFA uptake by cardiomyocytes (14), which impairs mitochondrial FA β-oxidation, leading to the accumulation of toxic lipid metabolites in the heart of patients with T2D compared with those with T1D (106, 107). It could be the underlying factor for this increased risk of CVD complications and mortality in T2D compared with T1D. However, in young patients with T1D, arterial stiffness is positively associated with glycated hemoglobin levels, disease duration, and insulin resistance. The arterial stiffness may predict cardiovascular events in asymptomatic individuals without overt CVD (108).

Endothelial dysfunction is a common complication in children and adolescents with diabetes, particularly T1D. Endothelial injury may signify the initial occurrence of vascular dysfunction, and hyperglycemia is probably the main driver of endothelial injury in pediatrics (109). A growing number of studies have illustrated a notable escalation in inflammation and endothelial dysfunction in children and adolescents with diabetes, which was then associated with an elevated cardiovascular risk (78). Mechanisms for the cardiovascular complications in adults with diabetes (14, 110-112) have been well addressed, namely, hyperglycemia increases ROS and advanced glycation end-products (AGEs) production, activates diacylglycerol (DAG)-protein kinase C (PKC) signaling pathway, hexosamine and polyol pathways, and affects the function of endothelial progenitor cells. The activation of the DAG-PKC signaling pathway contributes to the increased permeability of endothelial cells. Besides, the deposition of AGEs triggers vascular inflammation and leads to increased connective tissue crosslinking, fibrosis, and cardiac stiffness, thereby impairing diastolic relaxation. Endothelial dysfunction is a significant concern in pediatric patients with diabetes, and the early detection of endothelial dysfunction in children and adolescents with diabetes might be useful to prevent, or at least delay, cardiovascular complications (113-118).

## OXIDATIVE STRESS AND DIABETIC CARDIOVASCULAR COMPLICATIONS IN PEDIATRICS

There is wide evidence that cellular pro-oxidative status and proinflammatory environment are strong triggers in the pathophysiology of diabetes and cardiovascular complications (119, 120). Overproduction of ROS and RNS due to altered redox signaling in cells and tissues results in elevated cellular-oxidative stress. The excessive oxidative stress in the cell causes modifications of structural and functional proteins, DNA damage, endoplasmic reticulum (ER) stress, and lipid peroxidation (LP), leading to cellular dysfunction (118). Cellular dysfunction results in inflammation and cellular and mitochondrial dysfunction manifested by altered molecular pathways, inactivation of enzymes, mitochondrial respiratory chain dysfunction, and deregulation in cell cycle control. This results in overall decreased biological activity and impairment of the antioxidant systems (118). However, the underlying mechanism of oxidative stress in diabetic cardiovascular complications, particularly in pediatrics, has been somehow neglected. Therefore, in this section, the current study will discuss underlying mechanisms in the formation of oxidative species and understand the molecular mechanisms of oxidative stress in pediatric diabetes and pediatric diabetic cardiovascular complications.

### Oxidative Stress

ROS and RNS are classified into two groups of compounds namely, free radicals and nonradicals. Examples for the radicals include superoxide (O_2_^•−^), oxygen radical (O_2_^••^), hydroxyl (OH•), nitric oxide (NO•), alkoxy radical (RO•), and peroxyl radical (ROO•); however, other nonfree radicals can also be found, such as hydrogen peroxide (H_2_O_2_), ozone (O_3_), dinitrogen trioxide (N_2_O_3_), and peroxynitrite (ONOO−). ROS are essential for the maintenance of a homeostatic environment for cell survival, and different cellular processes, such as protein phosphorylation, defense mechanisms, activation of transcription factors, and apoptosis, depend on the cellular concentration of ROS (121). Therefore, ROS is important for survival and adaptation. For instance, superoxide anion and hydrogen peroxide are substantial factors that are involved in the developmental signaling transduction in the pancreatic β-cell’s control of insulin secretion (118, 122). Approximately 90% of endogenous ROS are produced in the mitochondria through electron leakage from the respiratory chain, resulting in the formation of superoxide. Several endogenous antioxidant enzymes neutralize the produced ROS. The main endogenous antioxidant enzymes are superoxide dismutase (SOD), catalase (CAT), and glutathione peroxidase (GPx). ROS can be neutralized or scavenged by other nonenzymatic molecules with free radical scavenging properties such as melatonin, vitamins, glutathione (GSH), and metallothionein (MT). Nevertheless, oxidative stress is characterized by the imbalance between the production of ROS/RNS and neutralization by endogenous antioxidant enzymes. Prolonged oxidative stress leads to various life-threatening pathological conditions such as aging, diabetes, heart diseases, cancer, autoimmune diseases, and neurological disorders (118, 122, 123).

### Oxidative Stress in Pediatrics

**Figure 2.**
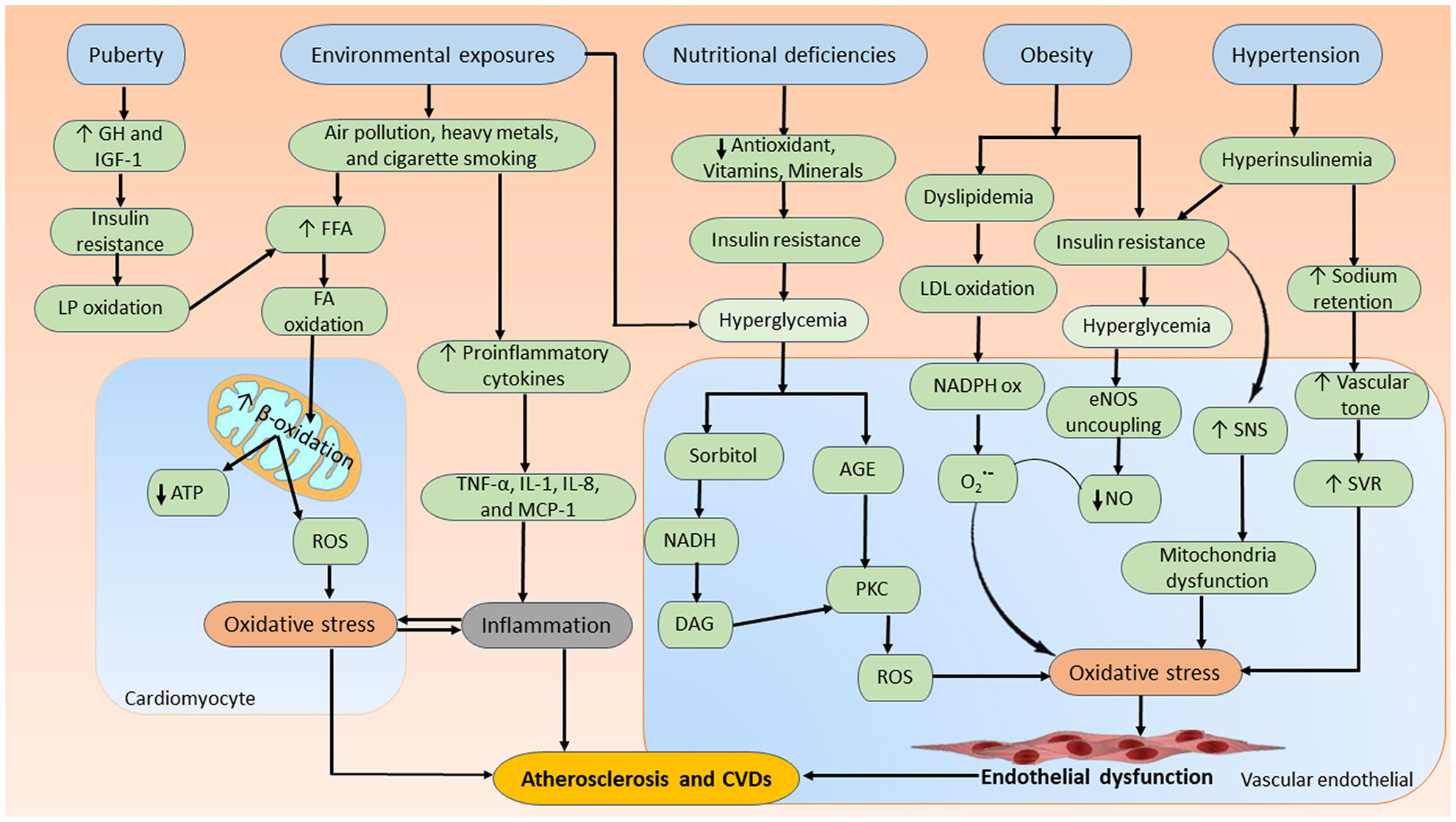
Main factors and their potential underlying signaling pathways that induce cardiovascular complications of diabetes in pediatrics. Nutritional deficiencies, puberty, environmental exposures, and metabolic disorders, such as obesity and high blood pressure (hypertension), can induce cardiovascular complications of diabetes in pediatrics including CVD, cardiac dysfunction, and atherosclerosis from a variety of molecular mechanisms. AGE, advanced glycosylation end-products; ATP, adenosine triphosphate; CVD, cardiovascular disease; DAG, diacylglycerol; eNOS, endothelial nitric oxide synthase; FFA, free fatty acids; GH, growth hormone; IGF-1, insulin-like growth factor 1; IL, interleukin; LDL oxidation, lipoprotein cholesterol oxidation; LP, lipid peroxidation; MCP-1, monocyte chemoattractant protein-1; NADH, nicotinamide adenine dinucleotide; NADPH, nicotinamide adenine dinucleotide phosphate; NO, nitric oxide; O_2_^•−^, superoxide; PKC, protein kinase C; ROS, reactive oxygen species; SNS, sympathetic nervous system; SVR, systemic vascular resistance; TNF-α, tumor necrosis factor-α.

Several studies have demonstrated the higher concentrations of oxidative stress markers in pediatric patients with diabetes compared with healthy controls (118, 124, 125). As mentioned earlier, oxidative stress is a main candidate accelerator of cardiovascular complications in patients with diabetes, independent of conventional risk factors. In pediatric patients with diabetes, several factors can contribute to the development of oxidative stress and excess ROS, including nutritional deficiencies, puberty, environmental exposures, and metabolic disorders such as obesity and high blood pressure (hypertension) (118, 126). These factors can induce cardiovascular complications of diabetes in pediatrics including CVD, cardiac dysfunction, and atherosclerosis from a variety of molecular mechanisms. Three potential underlying mechanisms are summarized in Fig. 2. Therefore, this section will discuss the following oxidative stress-associated factors that may be one key mechanism for pediatric diabetic cardiovascular complications.

#### Obesity.

However, hyperlipidemia, in conjunction with the global obesity epidemic, has emerged as the most prevalent, playing a key role in the development of CVD in individuals with diabetes (127). According to data from the National Health and Nutrition Examination Survey (NHANES), 20% of patients aged 12–19 yr show lipid disorders such as hyperlipidemia. Particularly, the category of children with obesity usually presents an even higher prevalence of hyperlipidemia, up to 42% (128, 129). This condition leads to a concomitant increase in the prevalence of CVD in childhood. Although these cardiovascular risk factors can appear at a young age, they tend to track into adulthood and determine a subsequent high risk for cardiovascular events in adult patients, as recently demonstrated by Jacobs et al. (130). Therefore, screening for hyperlipidemia is strongly recommended to detect high-risk children presenting with these disorders, as these patients deserve more intensive investigation and intervention.

On the contrary, obesity is the risk factor for hypertension, insulin resistance, and dyslipidemia (131) and has become an important health issue in childhood (132). This risk is more pronounced in youth with obesity and diabetes, as they may exhibit systemic inflammation and endothelial injury (133, 134), along with elevated levels of oxidative stress (135). The correlation among oxidative stress, obesity, and cardiovascular complications seems to be a consequence of low-grade inflammation (136). Low-grade inflammation is implicated in the pathogenesis of essential hypertension, with vascular inflammation occurring before systemic inflammatory changes. Evidence shows the presence of systemic oxidative stress in hypertensive children and adolescents, regardless of their body mass index, characterized by decreased nitrate levels and elevated LP end products. The ratio between LP and NO is directly associated with both systolic and diastolic blood pressures for the overall patient population (137). Oxidative stress is believed to be linked to the release of adipocytokines, which is associated with metabolic syndrome, obesity, and activation of the renin-angiotensin-aldosterone system. A study has indicated that oxidative stress is connected with organ injury, such as left ventricular hypertrophy and carotid intima-media thickness, in children with hypertension, along with metabolic abnormalities, adipose tissue volume, and insulin resistance (138). Leptin is one of the most known adipocytokines associated predictively with IL-6 in youths with the grade of obesity, which is higher in children with T2D (139). Moreover, oxidative stress may play a crucial role in pediatrics with obesity-associated hypercholesterolemia, demonstrated by elevated levels of adenine dinucleotide phosphate (NADPH) oxidase, along with the oxidized LDL-C levels, compared with a group of healthy children, children with only obesity, or children with only familial hypercholesterolemia. The association of multiple cardiovascular risk factors and NADPH oxidase is related to greater endothelial dysfunction and enhanced oxidative stress in children (140, 141), as shown in Fig. 2.

#### Hypertension.

High blood pressure (hypertension) is a complex disease in younger children that is often related to other health conditions, such as heart defects and hormonal disorders. Hypertension in children can also contribute to the acceleration of atherosclerosis and CVD originating from genetic predisposition and environmental factors (142). The prevalence of hypertension in children with T1D is reported to be between 6% and 16% (143) and is reportedly found in over two-thirds of patients with T2D (144). An example of the relationship between chronic inflammation and CVD was demonstrated in a cross-sectional study of children and adolescents who had dental caries-related primary hypertension. Among hypertensive patients with poor oral status, the intensive oxidation of several plasma substrates, increase in ROS, LP, and inactivation of prostacyclin and NO, and an imbalance in the total antioxidant capacity were noted (145). Decreased NO bioavailability is associated with atherogenesis and can participate in enhanced cell adhesion, proliferation, vasoconstriction, and the generation of atherosclerotic lesions (146). On the contrary, diabetes through various mechanisms affects all the components of blood pressure, namely, cardiac output and systemic vascular resistance (SVR). Increased sodium retention in the diabetic state leads to an expanded blood volume and cardiac output, whereas activation of the sympathetic nervous system in the diabetic state leads to vasoconstriction and, hence, increased SVR (147). Vascular tone, and thus SVR, is regulated by the local balance of paracrine, autocrine, endocrine, and autonomic mechanisms of vasodilation and vasoconstriction. Endothelial dysfunction is a maladaptive phenotype of abnormal vasoreactivity brought about by an imbalance of these factors. NO is produced constitutively by vascular endothelial cells via endothelial nitric oxide synthase (eNOS), which transfers electrons from a heme group to l-arginine, forming l-citrulline and NO (148). Taken together, diabetes induces oxidative stress and endothelial dysfunction, leading to hypertension through increased SVR. Subsequently, hypertension contributes to the acceleration of atherosclerosis and CVD (Fig. 2).

#### Inappropriate nutritional intake.

Children and adolescents often adhere to inadequate diets, which are characterized by insufficient consumption of fruits and vegetables and excessive intake of sugars and fats, which is associated with a defect antioxidant status (149, 150). A food insecurity or lack of dietary antioxidant intake is associated with an unfadable cardiometabolic profile in children and adolescents. The presence of dietary vitamins, specifically carotenoids and tocopherols, is of significant interest due to their bioactive properties as antioxidants and potential positive effects on adipogenesis, lipid, and glucose metabolisms (151-154). On the contrary, deficiencies in essential nutrients, such as vitamins and minerals, in children can contribute to insulin resistance. This can lead to elevated blood glucose levels. For instance, the association of CVD biomarkers with overall diet quality, as measured by the Healthy Eating Index-2015 (HEI-2015), and its dietary components in youth with type 1 diabetes was examined in youth with type 1 diabetes, in an 18-mo behavioral nutrition intervention trial. Dietary intake from 3-day diet records and CVD biomarkers were assessed at baseline, 6, 12, and 18 mo. Healthy Eating Index-2015 was not associated with CVD biomarkers, but whole grain intake or greater increase in whole fruit intake was associated with lower CVD biomarkers. In contrast, added sugar, saturated fat, and polyunsaturated fat were positively related to the CVD biomarkers, respectively. Therefore, findings suggest that the intake of specific dietary components, including whole grains, whole fruits, added sugar, and PUFA, may influence cardiometabolic health in youth with type 1 diabetes, independent of glycemic control (155).

#### Puberty.

The onset of puberty is another factor associated with elevated levels of oxidative stress in pediatric patients with diabetes (156). Indeed, puberty entails physiological insulin resistance and triggers hyperinsulinemia that influences oxidative stress and inflammation (157, 158). Nevertheless, most available studies have investigated individual cardiometabolic alterations in T1D or obesity, without considering important aspects such as puberty or studying its influence on their findings. Indeed, pubertal children have indicated higher levels of LP substances in plasma than prepubertal children (158). Moreover, puberty could accelerate cardiometabolic alterations, since it has been observed that puberty worsens endothelial dysfunction in youth patients with T1D (159). By the onset of puberty, there is a surge in GH and IGF-1, which increases insulin resistance (45), resulting in increased LP oxidation and increased FFA in the circulation. Besides, an increase in sex hormones during puberty, especially androstenedione, increases the acute insulin response, which is an independent predictor of T2D (47). Consequently, insulin resistance and T2D development led to increased FFA uptake by cardiomyocytes (14). Overall, these findings demonstrate that the onset of puberty influences insulin resistance and oxidative stress. This ultimately plays an important role in contributing to the development and progression of cardiovascular complications of diabetes in pediatrics (160).

#### Environmental exposures.

The prevalence of T1D and/or T2D in both children and adults is increasing worldwide but varies geographically (29-32), suggesting the etiological mechanisms of these differences related to both genetic and environmental factors (161, 162). The risks of chronic exposure to environmental elements including chemicals (163, 164) and heavy metals such as cadmium, inorganic arsenic, and lead for diabetes and diabetic CVDs have been proposed (7, 165, 166). Exposure to environmental contaminants as an endocrine-disrupting (ED) chemical might aggravate risk factors for CVDs, such as dyslipidemia and hypertension in individuals with diabetes (167, 168). Unhealthy water and diet, air pollution, tobacco smoke, dangerous chemical agents, and other environmental threats affect the health of children. Children’s distinctive metabolism, physiology, and developmental requirements make them more vulnerable than adults to pollutants, which can have a lifelong impact such as disease, disability, and early death. A cross-sectional study of Iranian children has shown childhood and adolescent exposure to arsenic, lead, and chromium can impact metabolic conditions and CVD markers (169), and the early exposure to environmental risk and ED like metals such as cadmium (170). Exposure to these environmental chemicals can induce oxidative stress and trigger inflammation, resulting in increases in blood pressure and insulin resistance. It also induces vascular injury and accelerates atherogenesis. Environmental chemicals can increase circulating FA oxidation, leading to an excess influx of FFAs into cardiomyocytes. The excess FFAs undergo β-oxidation, generating ROS and causing damage to various cellular components, including mitochondria. Mitochondrial damage, in turn, leads to a reduction in ATP production. On the contrary, exposure to environmental chemicals has been associated with an increase in the risk of dyslipidemia, atherosclerosis, and hyperglycemia in children (171, 172). Hyperglycemia is probably the main driver of endothelial injury in pediatrics and dyslipidemia may explain, in part, the increased incidence of CVD and atherosclerosis (109).

Environmental exposures in early life that contribute to T1D or children’s insulin resistance and obese risks may be also related to maternal exposure to these endocrine-function-like chemicals and metals on the fetus during pregnancy, neonatal factors, or later effects during infancy and early childhood (173). For example, analyses of T1D incidence of 0–9 yr individuals with diabetes over the period from April 2000 to March 2011 (*n* = 13,948) identify a range of demographic and environmental factors associated with T1D in children in England (174). A similar conclusion regarding the relationship between the total air pollutants emitted and the incidence of T1D and T2D in the Russian Federation was also reported (175).

## THERAPEUTIC STRATEGIES FOR PREVENTION AND TREATMENT OF DIABETIC CARDIOVASCULAR COMPLICATIONS IN PEDIATRICS

In pediatrics, oxidative stress and low-grade inflammation lead to impaired insulin secretion and insulin resistance, resulting in diabetes. Excess ROS influences both the formation of diabetes and its cardiovascular complications. However, the protective mechanism against oxidative stress and inflammation, and therapeutic strategies for prevention and treatment of pediatric diabetic cardiovascular complications should be addressed in future studies. Here, in this section, the current study highlighted the important strategies to prevent and treat diabetic cardiovascular complications in pediatrics. Therapeutic strategies for prevention and treatment of diabetic cardiovascular complications in pediatrics are summarized in Table 1.

### Lifestyle Strategy

Regular physical activity during early stages of life plays a crucial part in the diabetes management strategy in pediatrics contributing to various advantages such as weight loss and improvement in body mass index (BMI). This results in a reduction in oxidative stress and inflammatory biomarkers, increased tissue sensitivity to insulin, as well as the improved quality of life of patients with diabetes (49). Indeed, regular exercise has been shown to increase the release of anti-inflammatory cytokines, and they lead to a reduction in proinflammatory cytokines, such as TNF-α and IL-1, IL-6, IL-8, and C-reactive protein (CRP), which play critical roles in the pathogenesis of diabetes (48). Besides, intensive training improves glycemic control and antioxidant potential including total antioxidant capacity (TAC), CAT, and manganese-dependent SOD (50). In normal-weight children and adolescents, physical activity also increases NO bioavailability (176).

Another consideration is cigarette smoke exposure in children, which remains highly prevalent. Cigarette smoking during childhood and adolescence causes significant health problems among young people (177). Tobacco smoke contains several harmful compounds that can cause diabetes and its cardiovascular complications through a mechanism of increased systemic inflammation and free radical production (178). Smoking may also contribute to the development of diabetes by increasing levels of inflammatory markers including IL-6 and CRP (179). Cigarette smoking also induces dyslipidemia by increasing plasma concentrations of TG coupled with a decrease in HDL and then with hyperinsulinemia and insulin resistance (51). Dyslipidemia and hyperinsulinemia in those who smoke are significantly associated with an increased risk of cardiovascular complications, particularly coronary heart disease (CHD) in both T1D and T2D patients (52). An effective education program for children to quit smoking gradually reduces the risk of developing diabetes or vascular complications. Therefore, more emphasis should be placed on stopping smoking in this high-risk group of patients with diabetes.

### Nutritional Strategy

Medical nutrition therapy is the cornerstone of diabetes treatment, support of which both Mediterranean (MED) and low-carbohydrate (LC) diets are potentially beneficial for glycemic control in youths with T1D. Based on a recent prospective interventional study (180), the change in micronutrient intake of youth with T1D before and after a 6-mo MED intervention were assessed for 20 adolescents, with a median diabetes duration of 9 yr. At 6-mo postintervention, they demonstrated the feasibility for the MED-based intervention in youth with T1D to improve monounsaturated fat intake and diastolic blood pressure without change of caloric intake, BMI, and HbA1c (180). Similarly, the rationale for the LC diet’s potential benefit for T1D may be because carbohydrates are the primary macro-nutrients that affect postprandial glycemic response. In addition, reducing dietary carbohydrate consumption lowers both the amount of insulin administered and fluctuations in blood glucose levels. To support this notion, several studies support the clinical recommendations for individuals who choose to adopt a low-carbohydrate diet (181). The most recent study directly compared MED and LC diets for 6 mo with the conclusion that both MED and LC diets improved glycemic outcomes in adolescents and youths with T1D, without increasing hypoglycemia or cardiovascular risk factors, indicating comparable safety and efficacy (182).

Nutrition might play an important role in low-grade inflammation in children with diabetes, contributing to the pathogenesis of atherosclerosis. Polyunsaturated fatty acids (PUFAs) particularly omega-3 intake have beneficial effects on human health, especially CVD, via the decreased production of inflammatory eicosanoids, cytokines, ROS, and the expression of adhesion molecules (53). Patients with T2D are at risk of accelerated atherosclerosis due to elevated rates of inflammation and increased oxidative stress. It has been demonstrated that adherence to a “methionine-restrictive” diet has been also reported to improve lipid metabolism, increase oxidative capacity, and decrease systemic inflammation (54).

It has been indicated that oxidative stress and decreases in antioxidant defense mechanisms increase in children with cardiovascular risk, whereas antioxidant treatment may decline diabetic cardiovascular complications (183). For instance, treatment with vitamin C blocked the acute hyperglycemic impairment of endothelial function in adolescents with T1D (184). Moreover, chronic intake of vitamin C in high doses (≥1 g/day) improves glucose metabolism and increases insulin sensitivity in patients with T2D (55). The supplementation of several antioxidant vitamins complex exerts a more potent synergistic protective impact compared with the consumption of individual vitamins alone (56). However, an anti-oxidative function of the vitamin complex takes place only at low doses while pro-oxidative action would happen, suggesting that vitamin complex can inhibit or activate oxidative stress and proinflammatory cytokines dependent on the dose levels (56). Therefore, therapy with vitamin supplementations may decrease oxidative stress and ROS production in patients with diabetes, leading to a decrease in diabetic cardiovascular complications in pediatrics.

Zinc is an essential trace element commonly found in red meat, poultry, and fish. Zinc is involved in many aspects of cellular metabolism as a component of many enzymes. The recommended dietary allowance of zinc for pediatrics is 2–11 mg/day by the Food and Nutrition Board (FNB) at the National Academies of Sciences, Engineering, and Medicine (185). Zinc plays a crucial role in insulin synthesis, storage, and secretion. Research has shown that zinc deficiency could increase the risk of insulin resistance, glucose intolerance, diabetes, atherosclerosis, and CAD. Zinc supplementation improves the apolipoprotein A1 and B levels, oxidized LDL, leptin, malondialdehyde, and CRP (57).

Flavonoids are widely found as secondary metabolites in fruits, plants, and vegetables, notably berries and citrus fruits, leafy vegetables, garlic, onions, and beverages such as tea and wine (186). The antidiabetic properties of certain flavonoids and flavonolignans have been widely reported, including hypoglycemic, antioxidant, and anti-inflammatory effects and their modulatory role in cell signaling and immune-inflammation responses (187). The cardioprotective effects of flavonoids have been demonstrated in diabetic animal models. For instance, the administration of quercetin at 50 mg/kg body wt daily for 6 wk was found to mitigate diabetes-induced vasoconstriction and lower the heightened blood pressure in diabetic rats (58). The cardioprotective effects of quercetin may be attributed to its anti-inflammatory effects exhibited via its inhibition of aortic NF-κB and reducing the serum level of both TNF-α and CRP (58). Curcumin is a lipophilic polyphenolic substance from the rhizome of an herb. It has been recognized for its notable role in the prevention and treatment of CVDs, primarily attributed to its antioxidant and anti-inflammatory properties (188, 189). Notably, curcumin has demonstrated antiatherosclerotic effects, which can be ascribed to its ability to reduce elevated plasma cholesterol levels, inhibit LDL peroxidation, and attenuate LP (59). The main flavonoids that have shown antidiabetic and cardioprotective function to prevent and treat diabetic cardiovascular complications are presented in Table 1.

### Pharmacological Therapy

Pharmacological therapy for children and adolescents (individuals <18 yr of age) remains a challenging issue since it cannot be simply following the routine care for the adults with diabetes. The epidemiology, pathophysiology, developmental considerations, and response to therapy in pediatric diabetes are often different from those of adult diabetes (Table 2). There are also differences in recommended care for children and adolescents with T1D and T2D (190).

Some patients fail to achieve appropriate glycemic control with dietary management and exercise alone and require pharmacological treatment to improve glycemia (194, 195). A variety of antidiabetic drugs are currently available for adult patients that can improve blood glucose levels, prevent acute and chronic diabetes complications, improve insulin sensitivity, and improve endogenous insulin release and glucagon and incretin physiology (194, 196). However, options for children and adolescents remain limited, and evidence of the efficacy and safety of the majority of available treatments in children and adolescents continues to restrict care delivery. Nevertheless, several clinical trials of newer antihyperglycemic drugs are ongoing in pediatric patients with diabetes.

Pharmacological treatment of T1D continues to be based on insulin, and the Diabetes Control and Complications Trial established the role of intensive diabetes management to reduce complications of T1D. In fact, recently, the intensive management to achieve glycemic control was also established as the goal of treatment for youth with T2D (197, 198). In addition, pediatric patients with T2D may be treated with a combination of lifestyle modifications and pharmacotherapy that includes both oral and injectable diabetes medications, such as insulin and those that are able to reduce oxidative stress-induced cardiovascular complications; however, in some cases, pediatric diabetes requires other medical interventions (199).

To date, statins are considered as the first therapeutic approach due to their efficacy, safety, and cheapness (63). These drugs are inhibitors of the enzyme 3-hydroxy-3-methylglutaryl-CoA reductase (HMGCR), the primary player in hepatic cholesterol synthesis. Statins have been proven to reduce several blood lipids, namely by acting on TG, TC, and LDL-C blood levels, while also increasing HDL-C (200). Currently, seven commercially available statins have been approved for children and adolescents. Rosuvastatin is approved for use in children from the age of 7 yr; pravastatin and pitavastatin are useful in patients with 8 yr or older, whereas simvastatin, atorvastatin, lovastatin, and fluvastatin may be used in patients from the age of 10 yr (64). Moreover, their administration decreases the risk of CVD in children with both hypercholesterolemia and secondary hyperlipidemia, even if lipid levels are not substantially controlled (201).

Sulfonylureas can also be used to treat diabetes and function by activating the β-cells to secrete insulin. Although sulfonylureas are not generally used for the pediatric population, they have been known to be safe for children (202). The specific types of sulfonylureas that have been studied in children are glimepiride and glipizide. The caveats of sulfonylureas are usually weight gain and hypoglycemia. Clinical studies using glimepiride in children showed that it was equally effective in treating T2D as metformin (65).

Numerous preclinical studies have assessed the efficacy of blunting inflammatory cell trafficking with the use of pharmacological inhibitors to prevent the development of cardiovascular complications (14). Inhibitors of toll-like receptor 4 (TLR4) signaling and pro-inflammatory cytokines have been assessed in preclinical studies to prevent diabetic complications (Table 2). Suppression of TLR4 signaling with matrine or triptolide improves cardiac function and reduces collagen deposition in rat models of DCM (66, 67). In addition to strategies targeting FA oxidation to treat diabetic cardiovascular complications, insulin sensitizers that increase glucose oxidation and decrease lipid metabolism have also been assessed in preclinical models (14). GLP1 is an incretin that can reduce blood glucose levels in a glucose-dependent manner by increasing insulin secretion (68). The GLP1 analog exendin 4 and the dipeptidyl peptidase-4 (DPP4) inhibitor saxagliptin prevent the development of DCM via ameliorating lipotoxicity in a mouse model of T2D (69). However, a clinical study has recently indicated that the addition of metformin to insulin therapy did not significantly improve glycemic control in children with T1D but did provide a modest reduction in total daily insulin dose and BMI (71).

## CONCLUSIONS

Substantial evidence suggests that the early onset of T1D and T2D and prolonged exposure to these metabolic abnormalities amplify long-term cardiovascular complications in the pediatric population. Regarding this, cellular pro-oxidative status and proinflammatory environment are strong triggers in the pathophysiology of diabetes and cardiovascular complications in pediatrics. In fact, overproduction of ROS and RNS due to altered redox signaling in cells and tissues results in elevated cellular oxidative stress. Oxidative stress is connected with organ injuries, such as left ventricular hypertrophy and carotid intima-media thickness, in children with hypertension, along with metabolic abnormalities, adipose tissue volume, and insulin resistance. Overall, diabetes in pediatrics induces oxidative stress and endothelial dysfunction, leading to hypertension through increased systemic vascular resistance. Subsequently, hypertension contributes to the acceleration of atherosclerosis and CVD. Nutritional deficiencies, lifestyle habits, and environmental factors can exacerbate oxidative stress and cardiovascular risk in pediatric patients with diabetes. Antioxidant treatments, including vitamins, zinc, and flavonoids, have shown potential in reducing oxidative stress and improving cardiovascular health in individuals with diabetes. Regular physical activity and a healthy diet are crucial in diabetes management in children.

There are still many open questions regarding the underlying mechanisms of oxidative stress in diabetic cardiovascular complications in pediatrics and the precise role of lifestyle habits and nutrition in the development of pediatric diabetic cardiovascular complications. Therefore, further research is necessary to understand their contribution to pathophysiology as well as possibilities to modulate them as a therapeutic strategy.

The only treatment for T1D remains insulin replacement, delivered as multiple daily injections or by an insulin pump (198). Recent advances in technology include the hybrid closed-loop systems that combine data from a continuous glucose monitor and an insulin pump to provide real-time insulin dose adjustments. However, an increase in the time that the blood glucose levels are in the target range has been seen more consistently. This area warrants further evaluation for potential impact on long-term effects on cardiovascular complications.

The impact of hybrid closed-loop systems on cardiovascular complications and glycemic control in youth with T2D has not been sufficiently evaluated, though they are used clinically. Pharmacological treatment options for T2D have expanded in recent years and now include insulin, metformin, glucagon-like peptide-1 (GLP-1) agonists, and sodium-glucose cotransporter 2 (SGLT2) inhibitors. SGLT2 inhibitors decrease the risk of cardiovascular complications in adults with diabetes (203), whereas it is not known if they have similar effects in youth with diabetes.

In addition to pharmacotherapy, treatment includes lifestyle interventions with dietary changes and exercise. Weight loss is also a focus of treatment, including bariatric surgery. Bariatric surgery in teens has been demonstrated to result in high rates of remission of T2D (95%) within 3 yr after surgery (204).

In summary, children and adolescents with diabetes, regardless of type, have many features similar to those seen in adults, but there are some distinct differences (Table 2). Although pharmacological therapy focused on glucose management remains the primary focus for the treatment and management of children and adolescents with either T1D or T2D, innovative, adjuvant strategies that account for the complex interplay between oxidative stress, metabolic disorders, and cardiovascular health are urgently needed. By developing targeted therapies and preventive measures, healthcare providers can better address the rising incidence of diabetes-related complications in children and adolescents. Continued collaboration between researchers, clinicians, and public health officials is vital to effectively tackle this growing public health challenge.

## Figures and Tables

**Table 1.** Therapeutic strategies for prevention and treatment of diabetic cardiovascular complications in pediatrics

Strategy	Details	Signaling Pathways/Related Mechanisms	References
Lifestyle strategy	Physical activity	Decrease proinflammatory cytokines such as TNF-α, IL-1, IL-6, IL-8, and CRP	(48)
		Reduce ROS production, increase antioxidant potential, and enhances cellular sensitivity to insulin	(49)
	Intensive training	Improves glycemic control and antioxidant potential including TAC, CAT, and Manganese-dependent SOD	(50)
	Stopping smoking	Reduce TG and increase HDL	(51)
		Inhibition of hyperinsulinemia	(52)
Nutritional strategy	Polyunsaturated fatty acids	Decrease production of inflammatory eicosanoids, cytokines, and ROS	(53)
	Methionine-restrictive diet	Improve lipid metabolism, increase oxidative capacity, and decrease systemic inflammation	(54)
	Vitamin C	Improve glucose metabolism and increase insulin sensitivity	(55)
	Vitamin complex	Decrease the production of IL-4 and TNF-α and increase the production of IL-6	(56)
	Zinc	Contribute to improve the apolipoprotein A1 and B levels, oxidized LDL, leptin, malondialdehyde, and CRP	(57)
	Quercetin	Inhibition of aortic NF-κB and interference in NF-κB signaling	(58)
	Curcumin	Reduce elevated plasma cholesterol levels, inhibit LDL peroxidation, and attenuate LP	(59)
	Resveratrol	Promotes NO synthesis and inhibits endothelial dysfunction	(60)
	Genistein	Reduction in TNF-α, CRP, and TGF-β-1, and amelioration of the ultrastructural degenerative changes in the cardiac tissues	(61)
	Luteolin	Prevention of cardiac fibrosis, hypertrophy, and dysfunction in diabetic mice	(62)
Pharmacological therapy	Statins	Inhibitor of the enzyme HMGCR	(63, 64)
	Sulfonylureas (glimepiride)	Activation of β-cells to secrete insulin	(65)
	TLR4 signaling inhibitors	Improve cardiac function and reduce collagen deposition in rat models	(66, 67)
	GLP1	Reduce blood glucose levels in a glucose-dependent manner by increasing insulin secretion	(68)
	DPP4 inhibitor	Prevent the development of DCM via lipotoxicity ameliorating in a mouse model of T2D	(69)
	Sitagliptin	Decrease in oxidative stress and inflammatory markers such as nitrotyrosine, IL-6, and IL-18	(70)
	Metformin	Modest reduction in total daily insulin dose and BMI in children	(71)

BMI, body mass index; CAT, catalase; CRP, C reactive protein; DCM, diabetic cardiomyopathy; GLP1, glucagon-like peptide-1; HDL, high-density lipoprotein; LDL, low-density lipoprotein cholesterol; IL, interleukin; LP, lipid peroxidation; NO, nitric oxide; ROS, reactive oxygen species; SOD, superoxide dismutase; TAC, total antioxidant capacity; TG, triglycerides; TGF-β-1, transforming growth factor-beta-1; TNF-α, tumor necrosis factor-α.

**Table 2.** Distinct features of diabetes and related facts between children and adults

	Children	Adults
Types of diabetes	T1D as predominant type, ~23/100,000 (2017–2018) T2D: ~17/100,000 (2017–2018), which is increasing at ~3% yearly	T2D as the predominant form T1D prevalence (T1D: 1%–5%) increase, but the incidence and prevalence of T1D at older ages vary across the world, partly due to different definitions as well as population characteristics
Age of onset	T1D: 0–4 ≤ 15–19 < 5–9 < 10–14 T2D: 10–14 < 15–19	T1D: 30–59 > 18–29 > 60 – older T2D: 45–59 > 30–44 or 60 – older > 18–29
Sex	Boys > girls	
Obesity	Not common before, but gradually increases in the last two decades	Predominant
Diagnostic time of diabetes	Diagnosed suddenly due to acute symptoms such as excessive thirst, urination, weight loss, fatigue, and blurred vision. Diagnosis may happen during an emergency if diabetic ketoacidosis occurs	T2D is often diagnosed later in life, sometimes asymptomatically, through routine blood tests or when complications have already begun to develop (e.g., neuropathy, cardiovascular disease)
Key etiologies	Predominant autoimmune disease for T1D2) Less effects of lifestyle, environmental exposure, food insecurity on T1D, but may significantly on T2D	Autoimmune disease is not for T2D, but may exist in the individuals with T2D and T1D Significant effects of lifestyle, environmental exposure, unhealthy food on T2D
Features regarding insulin or β-cells	Insulin deficiency; require lifelong insulin therapy for survival Insulin needs can vary significantly throughout childhood and adolescence due to growth, hormonal changes, and activity levels In children with new onset T1D, younger age and male sex were associated with findings suggestive of more rapid and aggressive T1D preclinical course, including poorer β-cell function, and distinct islet autoantibody profiles Youths with T2D show a more rapidly progressive decline in β-cell function and accelerated development of diabetes complications	Insulin resistance T2D may not initially need insulin therapy May manage the condition with oral medications, lifestyle changes, and sometimes insulin therapy as the disease progresses
Growth concern	The presence of diabetes can interfere with normal growth and development. Poor blood sugar control can lead to poor growth, delayed puberty, or weight problems.	However, growth and development are typically not a concern
Cardiovascular complications and treatment		
Complications	Although children with diabetes are at risk for long-term complications, these may not be as pronounced in the early years of life. However, poor control during childhood can lead to more severe complications later in life, such as retinopathy, nephropathy, and cardiovascular disease The younger an individual is at diagnosis of T1D, the higher the risk of atherosclerosis and CVD For those with T2D, the onset of complications is earlier than seen in adults	In adults, diabetes can lead to complications such as cardiovascular disease, neuropathy, or kidney problems Adults are more likely to experience complications such as heart disease, stroke, kidney disease, nerve damage, and eye problems, especially if their diabetes has been poorly controlled over a long period
Treatment and monitoring	Treatment regimens often involve more frequent monitoring of blood glucose levels, adjusting insulin doses, and counting carbs Children may also have to balance school, extracurricular activities, and social situations, which can make diabetes management complex Devices like continuous glucose monitors and insulin pumps are commonly used	Adults may have a more stable routine for managing diabetes, especially if it is T2D. However, they also need to manage their condition in the context of work, family life, and other responsibilities
Diet and exercise	Encouraging healthy eating and physical activity can be more challenging due to children’s food preferences and activity levels. Children may also face peer pressure regarding their diabetes management	Adults may control over their diet and lifestyle, but work, stress, and other commitments also impact their ability to maintain healthy habits. Diabetes education and lifestyle changes are essential to manage T2D

CVD, cardiovascular disease; T1D, type 1 diabetes; T2D, type 2 diabetes. The contents are based on the information from all references cited in this review, but mainly from the references of Refs. 74-76, 190-193.
